# Fighting Ebola: A Window for Vaccine Re-evaluation?

**DOI:** 10.1371/journal.ppat.1006037

**Published:** 2017-01-12

**Authors:** Keith J. Chappell, Daniel Watterson

**Affiliations:** School of Chemistry and Molecular Bioscience, The University of Queensland, St Lucia, Australia; University of Pittsburgh, UNITED STATES

The unprecedented magnitude of the 2014/2015 Ebola virus (EBOV) outbreak in West Africa prompted the fast-tracking of experimental live replicating recombinant vaccines into clinical safety trials and field deployment. The epidemic was by far the worst crisis caused by any Filovirus, infecting ten times more individuals than all previous outbreaks combined. The outbreak was eventually brought back under control via methods of containment, including patient isolation, contact tracing, and safe burial practices. This achievement was made possible by the unwavering determination of countless volunteers under the guidance of the World Health Organization and multiple other aid organizations.

At the height of the West African crisis, it was unknown whether standard containment protocols would be enough to stem the epidemic. In the face of the looming crisis, the decision was made to accelerate the delivery of vaccine candidates and therapeutic antibody treatments, previously at an early stage of development, into clinical safety trials and field deployment. While localized infections still continue to appear and require continued vigilance, the immediate threat has now been brought under control. As a result, we are now presented with a unique window of opportunity to reassess these vaccines and decide whether they are optimal to combat future outbreaks.

To date, seven vaccine candidates have entered clinical safety trials (reviewed [1–3]). Of these, three have progressed to efficacy trials after completion of Phase I trials and include ChAd3-ZEBOV, Ad26-EBOV/MVA-EBOV, and rVSV-EBOV. ChAd3-ZEBOV and Ad26-EBOV/MVA-EBOV are both adenovirus-based vaccines and contain the EBOV glycoprotein (GP) in place of the native adenovirus early region 1. This region is essential for virus replication, and the genetic substitution at this site renders both ChAd3 and Ad26 viruses nonreplicating [4,5]. While this feature provides additional safety, immunogenicity issues have been observed, necessitating high vaccine doses and multiple immunizations. In the case of Ad26-EBOV, a heterologous booster regime utilizes MVA (modified vaccinia Ankara) to deliver a subsequent dose of Ebola GP. Nevertheless, both candidates have had promising results in both nonhuman primate (NHP) models and human trials [4–7].

**Fig 1:**
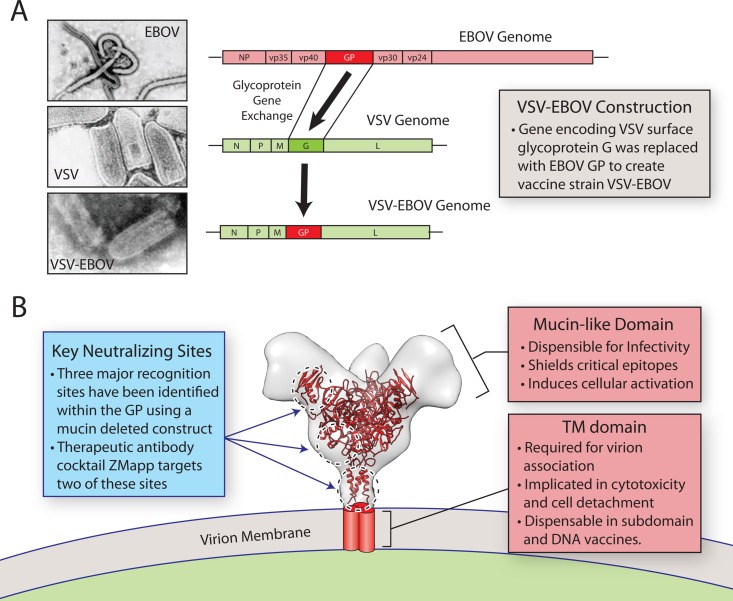
The live attenuated VSV-EBOV vaccine and EBOV GP functional attributes. **(A)** To create an attenuated vaccine for EBOV, the glycoprotein of VSV was replaced with that of EBOV GP. The resulting virus, rVSV-EBOV, forms bullet-shaped particles similar to VSV (Transmission electron microscopy analysis left panels [22,34,35]) rather than the long filaments usually observed for wild-type EBOV. EBOV GP is present on the surface of the chimeric virus in place of VSV G. **(B)** Recent research has revealed that the EBOV GP (known atomic structure presented in red—PDB-5JQ3 [36]—and cryo-electron tomographic structure of the complete form in outline—EMD-6003 [37]) contains three critical neutralization sites found at the glycan cap, GP1/2 interface, and the stem region, respectively [32,38]. Effective vaccine candidates would be expected to elicit a strong response to these epitopes. The mucin-like domain, which projects from the top of the GP trimer, acts as a shield to prevent immune recognition of neutralization sites [19] and has also been observed to initiate cell activation and the production of inflammatory cytokines in vitro [12]. The transmembrane (TM) domain tethers the GP trimer to the viral membrane and has been implicated in endothelial cell disruption [15]. The adverse effects of the mucin-like domain and the TM domain could be eliminated in future DNA or subunit vaccine lacking these regions.

The furthest progressed of the vaccine candidates is a live replicating recombinant virus based on the backbone of a vesicular stomatitis virus (rVSV-EBOV, Merck). This approach includes a functional full-length EBOV GP that is incorporated in place of the native VSV Glycoprotein (G) (Fig 1A). This vaccine had previously been shown to be protective in NHPs and was shown to be effective in humans in a delayed deployment efficacy trial conducted in Guinea at the tail end of the 2014/2015 outbreak [8].

The results of the delayed deployment efficacy trial led to rVSV-EBOV being widely reported as 100% effective. However, restricted to a short study window of only 11 days (10 and 21 post-vaccination), these encouraging results should be viewed with caution. Promisingly, no new cases were identified in the vaccinated population during the study window, compared to 16 cases in the population yet to receive the vaccine. However, two cases of suspected EBOV disease were reported within the vaccinated population at day 24 and 38 post-vaccination [8]. Careful evaluation of these events and any additional putative cases found to have occurred after the study window will shed light on the long-term efficacy of the vaccine approach. Surprisingly, while the interim results from this trial were reported in August 2015 [8], the final results are still yet to be released (as of October 2016), so it is currently unknown whether these suspected cases were later verified or whether any additional cases amongst vaccinated individuals have been detected.

In addition to this trial, four separate Phase I safety trials for rVSV-EBOV were completed, and the findings have been recently reported [9]. These trials were conducted in Geneva (Switzerland), Hamburg (Germany), Kilifi (Kenya), and Lambaréné (Gabon), using different doses of rVSV-EBOV. In the Geneva trial, which administered the highest dose, 11 cases of arthritis were identified (22% of participants), forcing a temporary hold. Joint pain and/or arthritis were also reported amongst participants at other trial sites, although cases were less frequent and predominantly less severe. In four participants from the Geneva trial, pain persisted between 2 and 6 months and a further two participants reported reoccurring joint pain after 2.5 or 4 months. In one participant with arthritis, rVSV RNA was detected in synovial fluid collected by knee arthrocentesis, indicating migration of the recombinant virus into the joint capsule. Nonserious adverse reactions that were frequently reported in all four safety trials included fever (20%), chills (29%), myalgia (45%), arthralgia (15%), headache (48%), and fatigue (42%). Within the delayed deployment efficacy trial, a severe episode of febrile illness was reported in a participant at day 2 post-vaccination and was deemed to be a direct result of vaccination [8]; however, rates of nonserious adverse events have not been reported.

The underlying cause of these adverse reactions remains open to debate. The VSV backbone has previously been shown to have an acceptable safety profile in humans when used to present HIV *gag* [10], however, use in humans is limited, and backbone contribution to the observed reactogenicity for rVSV-EBOV requires further investigation. Of note, in vitro studies have previously linked EBOV GP with cell cytotoxicity, inflammation, and vascular leak, raising the possibility that it may produce similar effects within the context of the recombinant vaccine and contribute directly to the observed adverse reactions. Within GP, two regions have been linked with cytotoxicity; the highly glycosylated, mucin-like domain and the c-terminal transmembrane (TM) region (Fig 1B).

The mucin-like domain has been shown to be directly involved in the detachment of endothelial cells from blood vessels, leading to increased vascular permeability [11]. This domain has also been shown to be involved in the interaction between GP and the pathogen-associated molecular pattern (PAMP) receptor, Toll-like receptor 4 (TLR4). TLR4 binding leads to cellular activation and the potent induction of inflammatory responses [12]. Given TLR4 activation has been linked directly to vascular leak in other viral systems [13], a central role for this pathway in EBOV pathology remains an untested but likely hypothesis [14].

Beyond the mucin-like domain, TM region has also been implicated in cytotoxicity. Expression of full-length GP triggers the extensive formation of filaments at the plasma membrane, followed by cellular detachment. The same effect is seen when only the C-terminal GP2 subunit is expressed but can be partially reversed by mutation within the TM region [15]. Additionally, it is worth noting that a significant portion of GP is shed from the infected cell surface through the activity of a metalloprotease, tumor necrosis factor α-converting enzyme (TACE). The extent to which this occurs upon vaccination with rVSV-EBOV has not been specifically addressed and could be optimized in future vaccines [16]. Such optimization could also provide important insights into EBOV pathogenesis as the shed form of GP has been specifically implicated in vascular permeability [17], while the membrane-anchored full-length form has been implicated in cytotoxicity [18]. Pertinent to vaccine design, neither the mucin-like domain nor the TM domain is essential for generation of a protective neutralizing immune response. Indeed, the epitopes targeted by the approved antibody cocktail treatment lie within the GP1/2 chalice and do not require the mucin-like or TM domains (Fig 1B). In fact, the mucin-like domain has been suggested to shield vulnerable epitopes from immune recognition, and the absence of this domain from virus-like particles has been shown to improve the neutralizing immune response upon immunization [19].

These studies highlight a possible causal relationship between discrete structural motifs within GP and EBOV pathogenesis, which raises the possibility that these effects may be minimized in future versions of EBOV vaccines. The current rVSV-EBOV and other live replicating recombinant vaccines utilize full-length EBOV GP; however, large alterations to GP structure appear a viable prospect, as previous work has demonstrated that the mucin-like domain is dispensable for the generation of replication competent virus [19,20]. Beyond live vaccines, other approaches, including DNA-based, subunit, and virus-like particles or other nonreplicating vaccines, are ideal vehicles for optimized GP delivery and could be designed to include known protective epitopes without undesirable masking or cytotoxic domains.

Of further concern is the theoretical potential for live replicating vaccines, such as rVSV-EBOV, to become transmissible in humans. While unlikely, this possibility is exemplified by outbreaks of vaccine-derived poliovirus [21]. The potential for transmission of chimeric recombinant vaccines represents a larger unknown compared to attenuated vaccines, and the repercussions from such an event could be far more dramatic.

Considering these risks, it is of concern that among vaccinated individuals, shedding of live recombinant virus has been recorded. Of 51 patients immunized with high doses of rVSV-EBOV in the Geneva trial, three developed maculopapular rash with vesicular lesions shown to contain VSV antigens. Genetic material from rVSV-EBOV could be detected in fluid released from vesicular lesions by qPCR out to 17 days after vaccination, and infectious virus was isolated from one individual 9 days after vaccination [9]. Direct contact with vesicular lesions could, therefore, theoretically facilitate transmission. This scenario could provide a window for the acquisition of mutations or genetic recombinations that could transform the recombinant virus from a vaccine into a pathogen in its own right.

While VSV infections in humans are rare and generally subclinical, incorporation of a potential virulence factor, such as EBOV GP, could promote unforeseen effects. EBOV GP is responsible for facilitating receptor binding and viral entry and, therefore, has the potential to alter cellular tropism. Indeed, altered tropism was observed and used to validate the incorporation of EBOV GP in the VSV system [22]. The reported vaccine-associated maculopapular rash observed in safety trials has not been reported for human infections with wild-type VSV and may be an indication that altered viral tropism is driving unique pathology in humans. Also of concern is the potential of GP-induced vascular leak, which may facilitate systemic dissemination of the live virus and increase transmission risk.

The level of rVSV-EBOV replication, and therefore risk of adverse effects, can be crudely adjusted by altering the vaccine dose. Ideally, a level could be identified that is both safe and protective. However, due to the natural variation in susceptibility between individuals, it may not be possible to define an appropriate dose that is both safe and protective in all individuals. This is of particular significance when considering potential use in children, pregnant women, and immunocompromised individuals in whom vaccination may trigger more severe adverse effects. Notably, clinical trials of the adenovirus-based vaccines Ad26-ZEBOV and CHAd3-EBOZ are being performed within these cohorts and may offer a safer alternative [23,24].

Despite these concerns, the rVSV-EBOV approach has several advantages, including a robust response that doesn’t necessitate a booster regime or adjuvant. This feature is especially valuable given the challenging logistics of vaccine delivery in endemic regions. In addition, rVSV-EBOV appears to have potential in both pre- and post-exposure settings. Data from post-exposure treatment in humans is thus far limited to one example, in which rVSV-EBOV was administered 43 hours after accidental needlestick exposure to EBOV [25]. In this instance, post-exposure treatment induced VSV viremia and anti-EBOV immunity, and the individual survived; however, it is unknown whether the initial exposure dose was life-threatening. Successful post-exposure treatment has also been demonstrated in NHP models [26,27], supporting the ongoing use of rVSV-EBOV in this manner wherever possible.

Consideration of potential risk versus benefit is an integral part of the implementation of any vaccination strategy. The high fatality rate associated with EBOV justifies the acceptance of a higher level of risk in the absence of a viable alternative. However, the higher potential for adverse reactions and the possibility of vaccine-derived infections warrant careful consideration when utilizing live replicating vaccines. Adverse reactions also have implications beyond the individual level, as community uptake may be reduced due to public concern about vaccine safety. As with rVSV-EBOV, other live replicating vaccines incorporating full-length EBOV GP and other viral backbones could be expected to suffer from similar complications. In parallel with clinical efforts, the recent outbreak also stimulated renewed basic research in Ebola, which is now translating into significant advances in the understanding of GP structure, function, and optimal neutralization sites at the molecular level [28–33]. These new insights should now be used to guide rational antigen design in order to minimize cytopathic effects and provide optimal protective efficacy. Now that the immediate threat from the West African EBOV outbreak has been brought under control, we have the capacity to establish an optimal vaccine strategy with the aim of delivering robust protection together with a stringent safety profile. We advocate for the careful assessment of the risks and benefits of utilizing live replicating vaccines for Ebola and for the expedition of subunit and other nonreplicating vaccines into clinical trials to provide alternatives for protection against Ebola.
